# Parametric Studies of Titania-Supported Gold-Catalyzed Oxidation of Carbon Monoxide

**DOI:** 10.3390/ma10070756

**Published:** 2017-07-05

**Authors:** Siewhui Chong, Thomas Chung-Kuang Yang

**Affiliations:** 1 Department of Chemical and Environmental Engineering, University of Nottingham Malaysia, Jalan Broga, 43500 Selangor, Malaysia; faye.chong@nottingham.edu.my; 2 Department of Chemical Engineering and Biotechnology, National Taipei University of Technology, No. 1 Zhongxiao East Road, Section 3, Da’an District, Taipei City 106, Taiwan

**Keywords:** gold, catalyst, TiO_2_, photodeposition, carbon monoxide

## Abstract

This paper remarks the general correlations of the shape and crystallinity of titanium dioxide (TiO_2_) support on gold deposition and carbon monoxide (CO) oxidation. It was found that due to the larger rutile TiO_2_ particles and thus the pore volume, the deposited gold particles tended to agglomerate, resulting in smaller catalyst surface area and limited gold loading, whilst anatase TiO_2_ enabled better gold deposition. Those properties directly related to gold particle size and thus the number of low coordinated atoms play dominant roles in enhancing CO oxidation activity. Gold deposited on anatase spheroidal TiO_2_ at photo-deposition wavelength of 410 nm for 5 min resulted in the highest CO oxidation activity of 0.0617 mmol CO/s.g_Au_ (89.5% conversion) due to the comparatively highest catalyst surface area (114.4 m^2^/g), smallest gold particle size (2.8 nm), highest gold loading (7.2%), and highest Au^0^ content (68 mg/g catalyst). CO oxidation activity was also found to be directly proportional to the Au^0^ content. Based on diffuse reflectance infrared Fourier transform spectroscopy, we postulate that anatase TiO_2_-supported Au undergoes rapid direct oxidation whilst CO oxidation on rutile TiO_2_-supported Au could be inhibited by co-adsorption of oxygen.

## 1. Introduction

The first publication on the oxidation of carbon monoxide (CO) using gold (Au) as catalyst was published by Bone and Andrew [1] in 1925. In the 1970s, Bone and Sermon [2] deposited gold on different supports—magnesium oxide, aluminum oxide and silicon oxide using thermal decomposition method, and demonstrated the hydrogenation of olefins at 100 °C. Later on, Huber et al. [3,4] showed that CO oxidation occurred using a matrix mixture consisting of oxygen, CO and Au, at temperature ranging from −263 °C (10 K) to −233 °C (40 K). Until 1987, Haruta et al. [5,6] used co-precipitation and precipitation method to deposit Au on metal oxide and showed that at room temperature of 24 °C, the oxidation activity was considerably high, and that approximately 5% of Au deposited on α-iron oxide showed the best oxidation activity. Since then, development of Au catalyst has become a popular topic and has been utilized in various applications, for instance, pollution control [7,8,9,10], and fuel cell [11,12,13].

Over the years, a large number of studies were carried out to improve the catalytic activity of Au nano-particles; the dominant factors can be divided into four categories [14]: type and morphology of support [15,16,17], the Au particle size [18], the nature of the active sites [19,20], and the preparation method including the pre-treatment [21]. The optimum Au particle size was suggested to be 3.5 nm [22], or less than 5 nm [5]. It was found that when the Au particle gets smaller, the number of edge or kink atoms increases, thus increasing the surface area/periphery of interaction with the support [23,24]. The amount of low-coordinated sites in Au was also shown to be a governing factor affecting the adsorption of CO on Au surfaces [25,26,27]. Cho [28] explained that the high activity of small metallic Au particles could be attributed to the small metal clusters displaying non-metallic behavior (quantum size effects), the presence of high densities of low coordinated atoms, excess electronic charge, and Au-support interactions (active perimeter sites). Unlike Haruta [29], Park and Lee [30] showed that higher calcination temperature increased the amount of Au^0^, but it was the presence of Au^3+^ that had enhanced CO oxidation activity. Various studies showed that the morphology and type of support play an important role in catalytic performance, for instance spindle-shaped Au/α-iron oxide was more superior than rhombohedral shape [31], and cerium oxide (CeO_2_)-titanium (TiO_2_) nanorods as support endowed the Au/CeO_2_-TiO_2_ catalyst with much higher activity than the CeO_2_-TiO_2_ nanoparticles [32]. Lopez et al. [27], on the other hand, concluded that the Au particle size was the determining factor affecting the catalyst’s activity (~1/d^3^ scaling law for the activity, with d being the particle diameter) instead of the support. Till now, due to the contradictory results, the support type and the nature of active sites remain to be the active research areas. In view of the lack of a generalization especially for TiO_2_, in this study, Au catalysts with different particle size and loading were synthesized by varying the photo-deposition condition, using anatase and rutile TiO_2_ as the supports, and taking into consideration the effect of the shape of support—cubic and spheroidal. This study aims to provide a general correlation relating CO oxidation activity with Au particle size (and specific surface area), Au loading, and Au^3+^/Au^0^ content, for Au-TiO_2_ synthesized using sol–gel and light emitting diode (LED) photo-deposition method.

## 2. Results

### 2.1. TiO_2_ Supports and Gold Deposition

**Figure 1:**
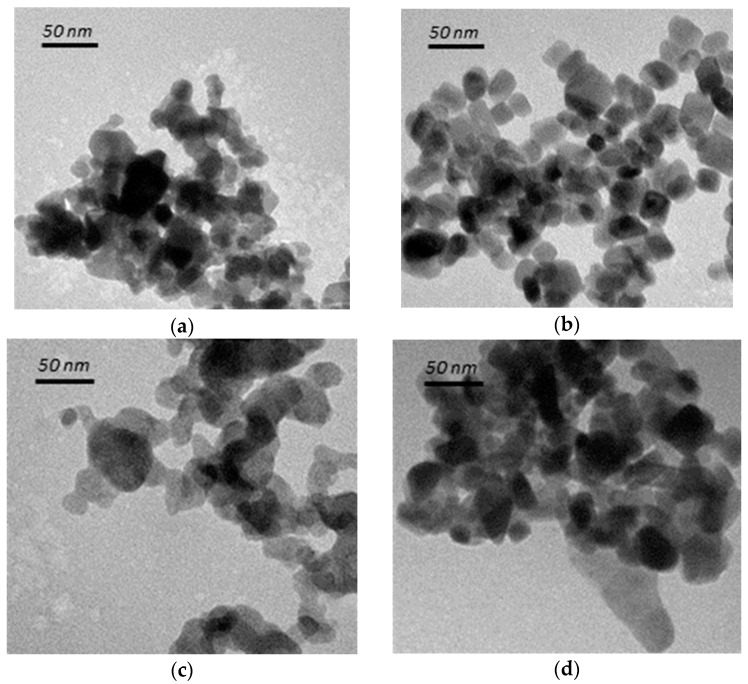
TEM images of (**a**) anatase spheroidal TiO_2_; (**b**) anatase cubic TiO_2_; (**c**) rutile spheroidal TiO_2_; and (**d**) rutile cubic TiO_2_.

Figure 1a,b shows that the as-prepared spheroidal TiO_2_ particles were a mixture of both spherical and rectangular shapes, with sizes of 20~35 nm; the anatase cubic TiO_2_ on the other hand, appears to be cubic in shape due to the use of tetrabutylammonium hydroxide (TBAH) agent as template, with sizes of 5–10 nm. For rutile TiO**_2_** as shown in Figure 1c,d, the less evident boundaries are probably due to crystal rearrangement at the boundary layers. The sizes of the as-prepared rutile TiO_2_ were in the range of 40–60 nm, about twice the sizes of anatase TiO_2_, due to the transformation of two or more of the anatase TiO_2_ particles under higher calcination temperature which favors particle growth.

**Figure 2:**
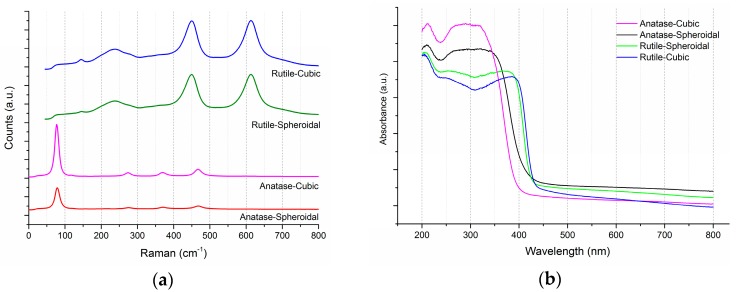
Raman and UV-VIS spectra of anatase and rutile TiO_2_ in both (**a**) cubic, and (**b**) spheroidal shapes. a.u.: arbitrary unit.

As shown in Figure 2a, the three characteristic E_g_ Raman modes are at 144 cm^−1^ (E_g(1)_), 197 cm^−1^ (E_g(2)_) and 639 cm^−1^ (E_g(3)_), as well as two B_1g_ Raman modes at 399 cm^−1^ (B_1g(1)_) and 519 cm^−1^ (B_1(g)_, A_1(g)_), thus confirming the anatase TiO_2_ structures. Rutile TiO_2_ on the other hand are confirmed by the indication of four characteristic modes at 143 cm^−1^ (B_1g_), 246 cm^−1^, 443 cm^−1^ (E_g_), and 609 cm^−1^ (A_1g_). The shapes of TiO_2_ had no significant impact on its phases or crystallinities. Figure 2b shows the UV-Visible (UV-VIS) spectra of anatase and rutile TiO_2_. In accordance to the transmission electron microscopy (TEM) images (Figure 1), the UV-VIS profile of rutile TiO_2_ was red-shifted, due to the larger particle size. The respective absorption thresholds of anatase spheroidal, anatase cubic, rutile spheroidal and rutile cubic are 408, 393, 422, 430 nm, corresponding to band-gap energies of 3.14, 3.26, 3.03, 2.98 nm.

**Figure 3:**
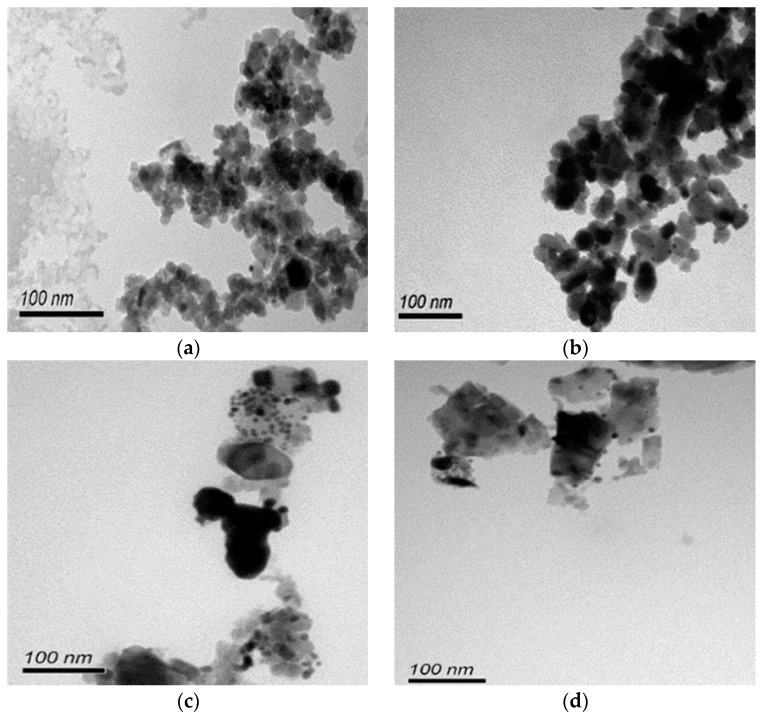
TEM images of (**a**) Au-anatase spheroidal TiO_2_; (**b**) Au-anatase cubic TiO_2_; (**c**) Au-rutile spheroidal TiO_2_; and (**d**) Au-rutile cubic TiO_2_, at photo-deposition wavelength of 365 nm.

**Figure 4:**
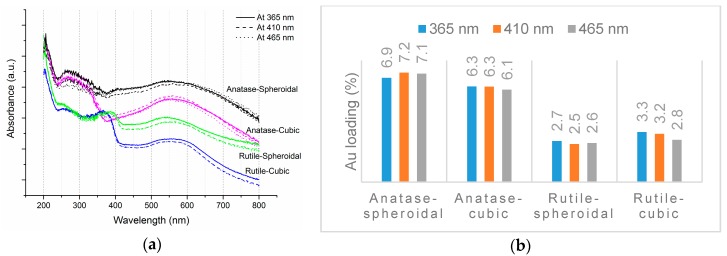
(**a**) UV-VIS spectra and (**b**) EPMA results of Au-TiO_2_.

**Figure 5:**
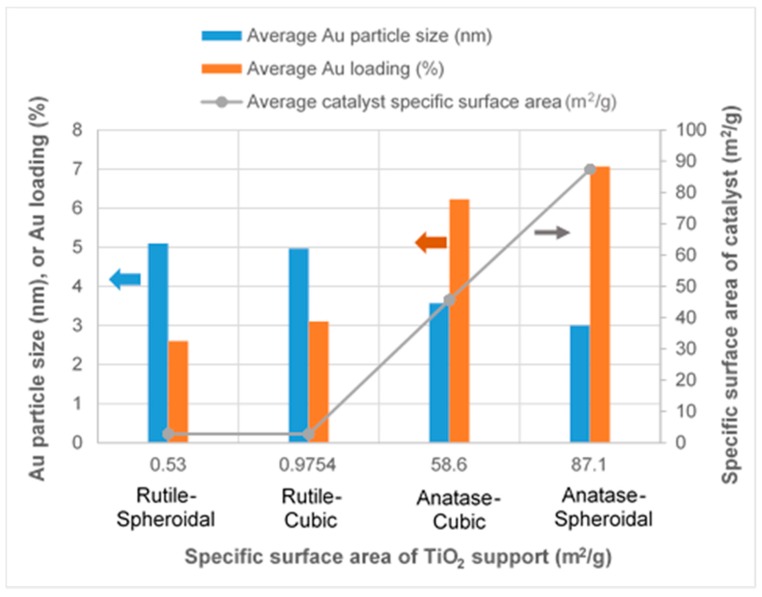
Effects of different TiO_2_ supports on Au particle size, Au loading and catalyst specific surface area.

As revealed by TEM images in Figure 3a,b, Au was evenly distributed on the anatase TiO_2_. Under illumination wavelengths of 365, 410, and 465 nm, the average sizes of the Au particles deposited on anatase spheroidal TiO_2_ were 3.2, 2.8 and 3.0 nm respectively; and those on anatase cubic TiO_2_ were 3.5, 3.6, 3.6 nm respectively. Au deposited on the rutile TiO_2_ supports, on the other hand, appeared to be uneven and agglomerating. The average Au particle sizes under illumination wavelengths of 365, 410 and 465 nm were respectively 4.9, 5.3 and 5.1 nm when deposited on rutile spheroidal TiO_2_, and 4.8, 4.9 and 5.2 nm when deposited on rutile cubic TiO_2_. As shown in Figure 4a, UV-VIS spectra display the presence of a broad peak at 550 nm for all Au catalysts, which could be ascribed to the local surface plasmon resonance (LSPR) [33,34] as a signature of the presence of Au^0^ [35,36]. Comparing the UV-VIS spectra of anatase and rutile TiO_2_ supports, anatase TiO_2_ resulted in higher LSPR peaks compared to rutile TiO_2_. As verified by electron probe micro-analysis (EPMA) in Figure 4b, the higher LSPR peak could be induced by the higher Au content. It is postulated that the higher gold loading and better gold dispersion on anatase TiO_2_ than rutile TiO_2_ could be due to the difference in the surface and textural properties [37], in which, as compiled in Table 1 and Figure 5, supports with higher surface areas and bigger pore volumes such as anatase TiO_2_ having specific surface areas of 58.6–87 m^2^/g, allowed better gold deposition and dispersion (smaller Au particle size and higher Au loading), in contrast to rutile TiO_2_ having much smaller specific surface areas in the range of 0.53–0.98 m^2^/g.

### 2.2. CO Oxidation

**Figure 6:**
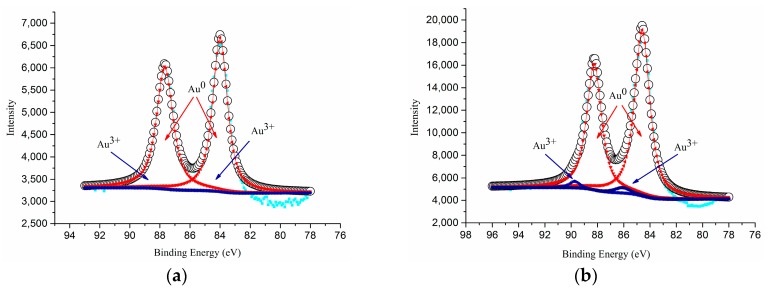
XPS spectra of (**a**) AS(410), and (**b**) RS(365).

Table 1 compiles the results of X-ray photoelectron spectroscopy (XPS), Brunauer, Emmett and Teller (BET), CO oxidation activity and CO conversion results of different catalysts. Among all, anatase spheroidal TiO_2_ support having highest specific surface area (87.1 m^2^/g) enabled highest gold loading (7.2%) and catalyst specific surface area (114.4 m^2^/g) which then enabled comparatively highest CO oxidation activity (61.7 × 10^−3^ mmol CO/s.g_Au_). As shown in Figure 6, the as-prepared Au-TiO_2_ catalysts contained only two gold states—Au^0^ and Au^3+^. The binding energies of Au^0^ (4f_5/2_), Au^0^ (4f_7/2_), Au^3+^ (4f_5/2_) and Au^3+^ (4f_7/2_) were respectively 86.8, 83.3, 88.4 and 84.8 eV. 

**Figure 7:**
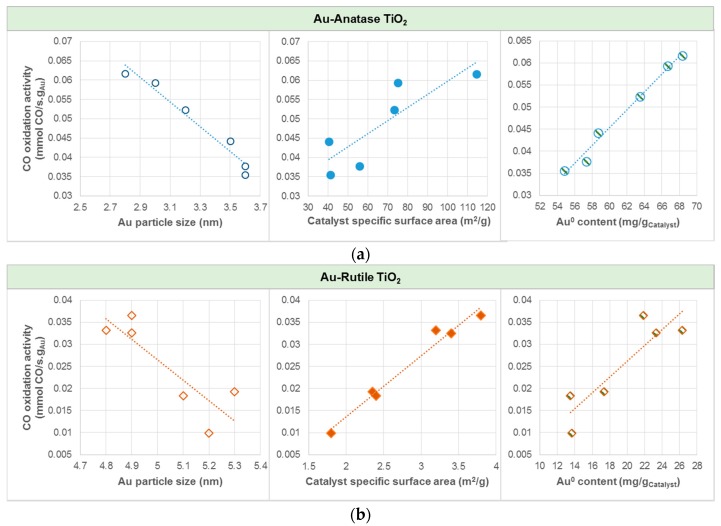
Effects of Au particle size, catalyst specific surface area, and Au^0^ content on CO oxidation activity for (**a**) Au-Anatase TiO_2_, and (**b**) Au-Rutile TiO_2_. Note: Au^0^ content in mg/g_Catalyst_ was calculated by the total amount of catalyst multiplied by the gold loading and the Au^0^ atomic percentage.

In average, gold catalyst supported on anatase was found to yield about three to six times higher CO oxidation activities than gold catalyst supported on rutile. Among the Au catalysts studied, AS(410) yielded the highest CO oxidation activity, corresponding to CO conversion of 89.5%. The plots of CO oxidation activity in Figure 7 show that, for both Au-anatase TiO_2_ and Au-rutile TiO_2_, higher oxidation activities could be attributed to the relatively smaller Au particle size and thus higher catalyst specific surface area, as well as a higher Au^0^ content.

It is apparent that Au particle size is a dominant factor affecting the catalyst surface area and Au loading. Smaller Au particles brings upon higher Au loading and thus higher catalyst specific area. Similar to the observations by [27], a property directly related to the size of the Au particles serves the vital role in the exceptional catalytic activity of Au catalyst, while other effects due to the support may have considerably smaller effect. It is thus postulated that small gold clusters in small support particles (more atoms having lower coordination number) induce stronger chemisorption [23,27,38], due to well-established varying electron density steps on model surfaces [39] and increase of the fraction of edge atoms [23,40].

As displayed in Figure 7, CO oxidation activity was found to be directly proportional to the Au^0^ content. Within the range of investigation, the higher the amount of Au^0^, the higher the CO oxidation activity. Catalyst with Au^0^ content of 68 mg/g_Catalyst_ (AS(410)) resulted in CO oxidation activity of 61.7 × 10^−3^ mmol CO/s.g_Au_ (89.5% conversion); whilst that with Au^0^ content of 14 mg/g_Catalyst_ (RC(465)) resulted in CO oxidation activity of 9.9 × 10^−3^ mmol CO/s.g_Au_ (5.6% conversion). Hodge et al. [41] nevertheless showed that the optimum Au^3+^/Au^0^ ratio was 1.5 for Au/iron-oxide catalyzed CO oxidation, and therefore generalizations remain an issue if it is to obtain for all catalysts, as much of the literature available are confined to specific types of catalyst. CO oxidations on anatase and cubic TiO_2_-supported Au catalysts in our case thus do not follow the Bond and Thompson model [7], which suggests that there is a need of a certain amount of Au^3+^ to serve as “chemical glue” binding the support with the gold particles for increased oxidation activity. Instead, as recognized by the correlation plots in Figure 6, the presented results are in good agreement with [42], which demonstrated that the CO conversion rate of Au particle (very close to pure Au^0^) was 100 times greater Au atom, i.e., oxidic Au, normalized to the number of Au atoms on the surface, for Au-ceria catalyzed CO oxidation at room temperature. Despite the important roles of those properties related to Au particle size, the difference in the CO oxidation of the anatase and rutile TiO_2_-supported Au catalysts can be explained using the DRIFTS analysis results.

**Figure 8:**
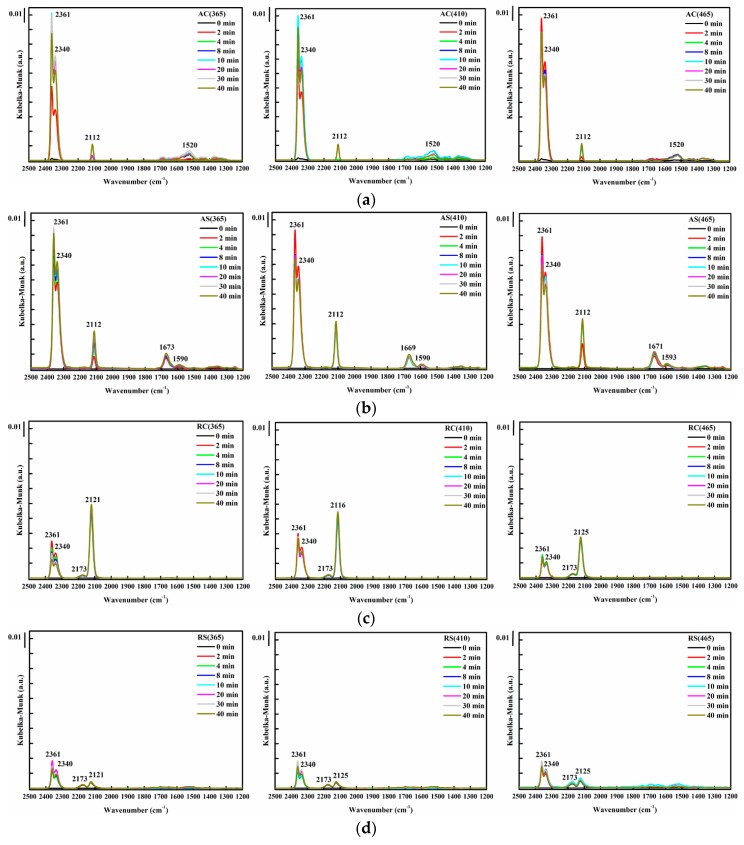
DRIFTS spectra of CO oxidation using Au catalyst on various supports: (**a**) anatase cubic TiO_2_; (**b**) anatase spheroidal TiO_2_; (**c**) rutile cubic TiO_2_; and (**d**) rutile spheroidal TiO_2_, photo-deposited at different wavelengths (from left to right, 365, 410 and 465 nm).

As seen in Figure 8a,b, for both cubic and spheroidal anatase TiO_2_-supported Au, approximately two minutes after feeding with CO, absorption peaks at 2112, 2340 and 2361 cm^−1^ appeared simultaneously. The former indicates adsorption of CO on Au^0^ [38,43], or more precisely, CO chemisorbed on the steps of small metallic Au particles [44], and the latter doublet indicates the formation of CO_2_ due to CO oxidation [44]. The byproduct formed was mainly monodentate carbonates (1520 cm^−1^) [45], bidentate carbonates (1669 cm^−1^) [46], and formates (1590 cm^−1^) [47]. The DRIFTS spectra of CO oxidation on rutile TiO_2_-supported Au catalysts on the other hand, revealed the noteworthy difference than those on anatase TiO_2_-supported Au catalysts. As shown in Figure 8c, two minutes after feeding with CO, unlike Au supported on anatase TiO_2_, no absorption peak at 2112 cm^−1^ was observed (which could also be overlapped); instead, the absorption peak at 2121 cm^−1^ appeared for RC(365) and RS(365); 2116 cm^−1^ for RC(410); and 2125 cm^−1^ for RC(465), RS(410) and RS(465), with the CO_2_ signals relatively smaller than anatase TiO_2_-supported Au catalysts. 

It was found that, the higher the 2112 cm^−1^ absorption peak in the DRIFTS spectra, the higher the CO oxidation activity, signaling the critical role of Au^0^. The reaction pathway of the CO oxidation on anatase TiO_2_-supported Au catalysts could be the rapid direct oxidation of CO at the surface of the metallic Au particles as suggested in [26,48]. On the other hand, the appearance of 2173 cm^−1^ absorption peak in all rutile TiO_2_-supported Au catalysts indicates CO adsorption on isolated carbonyl peroxo species (Au^δ+^-CO-O_2_^δ−^) [44] due to the higher amount of Au^3+^. This peak, however, did not seem to aid CO oxidation activity. Due to changes in the amount of charges, the 2112 cm^−1^ which was observed in anatase TiO_2_-supported Au blue-shifted to 2116 cm^−1^, signaling the CO-Au_s_^δ+^-O^δ−^ surface species [44,48]. The appearance of the absorption peak at 2121 or 2125 cm^−1^ in most rutile TiO_2_-supported Au catalysts signifies that electron transfer occurred from Au to the adsorbed oxygen molecules, i.e., CO was adsorbed on metallic Au interacting with a superoxidic or peroxidic oxygen molecular species [44], or the presence of CO-Au^δ+^ [49]. The low CO oxidation activity of rutile TiO_2_-supported Au catalyst was due to co-adsorption of oxygen (CO adsorption on pre-O_2_-adsorbed Au) which, to some extent, inhibited its CO oxidation activity [44,48,50].

## 3. Materials and Methods 

Fourteen milliliters of titanium (IV) isopropoxide (TTIP, Sigma-Aldrich, 99%, Taipei City, Taiwan, R.O.C.) were diluted with 25 mL distilled water for 30 min hydrolysis on a magnetic stirrer. The solution was then washed with distilled water in a centrifuge to remove contaminants and excess reactants before forming white precipitate. The precipitate was calcined at 400 °C for 5 h to obtain anatase spheroidal TiO_2_ particles. To obtain anatase cubic TiO_2_ particles, the white precipitate was mixed with 0.33 M TBAH (Honeywell Fluka, Shanghai, China) (40% in water) and stirred for 20 h before being poured to a Teflon autoclave for 12 h hydrothermal treatment at 200 °C. After that, the product was washed with ethanol and then calcined at 400 °C for 5 h. The same approach was carried out but both at calcination temperature of 1000 °C for 2 h to obtain rutile spheroidal and cubic TiO_2_ particles. 

For catalyst impregnation in the above support, 0.42 g of the above TiO_2_ was mixed with 25 mL distilled water and 8.5 × 10^−4^ M gold (III) chloride trihydrate (HAuCl_4_·3H_2_O, Sigma-Aldrich, 99%, Taipei City, Taiwan, R.O.C.). It was then adjusted to pH 9.5 using 1 M ammonium hydroxide (NH_4_OH, Sigma-Aldrich, 99%, Taipei City, Taiwan, R.O.C.) (28% in water). For photo-deposition of Au on TiO_2_, LED light tube of a fixed wavelength was placed on top of the beaker containing the solution. Three irradiation wavelengths were investigated—365, 410 and 465 nm (Thorlabs, Taipei City, Taiwan R.O.C.)—all with irradiation time of 5 min. After irradiation, the solution was washed with DI water in a centrifuge to remove solvents, the precipitate was then air-dried before being calcined at 300 °C.

The samples were analyzed with transmission electron microscopy (Hitachi H-7100, Taipei City, Taiwan R.O.C.) for displaying the morphology and size; Raman Spectrometer (DongWoo DM500i, Gyeonggi-do, Korea) and X-ray diffraction (Panalytical X’Pert PRO, Almelo, the Netherlands (with CuKα (λ = 1.5405 Å) radiation in the 2θ range from 20 to 70° at accelerating voltage of 45 kV and 40 mA) for determining the phase and crystallinity; UV-Visible spectroscopy (JASCO V-630, Gross-Umstadt, Gremany) for obtaining the band-gap energy. Electron probe micro-analysis (JEOL JXA-8200, Tokyo, Japan) for determining the Au loading; X-ray photoelectron spectroscopy (Kratos Axis Ultra DLD, Manchester, UK) for determining the elemental atomic ratios; and Physisorption BET (Micromeritics ASAP 2020, Norcross, GA, USA) for analyzing the surface area. In order to evaluate the CO oxidation efficiency, Fourier transform infrared spectroscopy (Perkin Elmer GX2000, Taipei City, Taiwan R.O.C.) (scanned in the region of 4000–1000 cm^−1^ at the resolution of 4 cm^−1^) coupled with DRIFTS sampling accessory (Harrick, Praying Mantis DRA, New York, NY, USA) was utilized to examine the CO oxidation activity using a catalyst loading of 60 mg, initial CO concentration of 2% in air, operating temperature at 20 °C, and a flowrate of 20 standard cubic centimeter per minute (SCCM). The gas outlet was connected to gas chromatography (Thermo GC Ultra Carboxen-1010, New York, NY, USA) for determining the outlet concentration. The CO oxidation activity was computed as the Change in CO concentration/(Reaction time × Catalyst weight). Au catalysts deposited on anatase cubic and anatase spheroidal TiO_2_ are denoted respectively as AC(X) and AS(X); and Au deposited on rutile cubic and rutile spheroidalTiO_2_ are denoted respectively as RC(X) and RS(X), with X being the photo-deposition wavelength used. 

## 4. Conclusions

Au was photo-deposited on anatase cubic, anatase spheroidal, rutile cubic, and rutile spheroidal TiO_2_ at various illumination wavelengths—365, 410 and 465 nm for 5 min—and examined for CO oxidation activity at room temperature. In contrast to anatase TiO_2_, due to larger pore volume and thus larger specific surface area, Au loading on rutile TiO_2_ was limited and the Au particles tended to agglomerate, thus affecting the particle size and amount of Au deposited. The average Au particle sizes on anatase cubic, anatase spheroidal, rutile cubic, and rutile spheroidal were respectively 3.6, 3.0, 5.0 and 5.1 nm, with Au loading respectively of 6.2%, 7.1%, 3.1% and 2.6%. XPS results revealed the presence of Au^3+^ and Au^0^. The highest CO oxidation activity (0.0617 mmol CO/s.g_Au_, corresponding to CO conversion of 89.5%) was obtained from anatase spheroidal TiO_2_ (410)-supported Au, with Au size 2.8 nm, Au loading 7.2%, specific surface area 114.4 m^2^/g, and Au^0^ content 68 mg/g_Catalyst_. Based on the results, we conclude that the surface and textural properties of the support, the contribution of gold states, and the nature of the metal/support interface (which accounts for the Au particle size, and Au loading) play critical roles in CO oxidation. We also postulate that Au deposited on anatase and rutile TiO_2_ supports result in different reaction schemes, in which a rapid direct oxidation takes place on the former, and co-adsorption of oxygen is likely to happen on the latter and, to some extent, inhibits the CO oxidation activity.

## Figures and Tables

**Table 1:** Au loading, Au particle size, X-ray photoelectron spectroscopy (XPS) analysis of Au^0^ and Au^3+^, Brunauer–Emmett–Teller (BET) and diffuse reflectance infrared Fourier transform spectroscopy (DRIFTS) results.

TiO_2_ Support	Sample Notation	Au Loading (%)	Au Particle Size (nm)	Au^0^ (Atomic %)	Au^3+^ (Atomic %)	Au^3+^/Au^0^ Ratio	Specific Surface Area (m^2^/g)	Conversion (%)	CO Oxidation Activity (mmol CO/s.g_Au_) × 10^−3^
**Anatase–Spheroidal**	-	-	-	-	-	-	87.1	-	**-**
	AS(365)	6.9	3.2	92	8	0.087	73.0	72.8	**52.3**
	AS(410)	7.2	2.8	95	5	0.052	114.4	89.5	**61.7**
	AS(465)	7.1	3.0	94	6	0.064	74.9	84.9	**59.3**
**Anatase–Cubic**	-	-	-	-	-	-	58.6	-	**-**
	AC(365)	6.3	3.5	93	7	0.075	40.3	56.1	**44.2**
	AC(410)	6.3	3.6	91	9	0.099	55.7	47.9	**37.7**
	AC(465)	6.1	3.6	90	10	0.111	41.0	43.7	**35.5**
**Rutile–Spheroidal**	-	-	-	-	-	-	0.53	-	**-**
	RS(365)	2.7	4.9	81	19	0.235	3.8	19.9	**36.6**
	RS(410)	2.5	5.3	69	31	0.449	2.4	9.7	**19.2**
	RS(465)	2.6	5.1	52	48	0.923	2.4	9.6	**18.3**
**Rutile–Cubic**	-	-	-	-	-	-	0.9754	-	**-**
	RC(365)	3.3	4.8	80	20	0.250	3.2	22.1	**33.2**
	RC(410)	3.2	4.9	73	27	0.370	3.4	21.0	**32.6**
	RC(465)	2.8	5.2	49	51	1.041	1.8	5.6	**9.9**
